# Follower arm muscle activity during the driver swing and its association with stroking performance in male golfers of different skill levels

**DOI:** 10.3389/fbioe.2026.1946349

**Published:** 2026-09-18

**Authors:** Chao Li, Heng Liu, Chao Liu, Baohua Liu, Zefeng Wang, Liang Chen

**Affiliations:** 1 School of Social Sports, Tianjin University of Sport, Tianjin, China; 2 School of Sport Training, Tianjin University of Sport, Tianjin, China; 3 China Institute of Sport Science, General Administration of Sport of China, Beijing, China; 4 Institute of Sport, Exercise and Health, Tianjin, China

**Keywords:** driver, follower arm, golf, stroking performance, surface electromyography

## Abstract

**Objective:**

This study aims to investigate the activation patterns of the follower-arm muscles during the driver swing in male golfers of different skill levels and to examine their associations with stroking performance parameters, with the goal of elucidating the functional role of the follower arm in the golf swing.

**Methods:**

Sixteen right-handed golfers (8 National Level 1 and 8 National Level 2 athletes) participated, and multiple valid swing trials were collected from each participant with each individual swing treated as a measurement unit. Surface electromyography (sEMG) signals were collected from the follower-side triceps brachii, biceps brachii, pronator teres, flexor carpi ulnaris, and extensor carpi radialis brevis. The swing cycle was divided into five phases (P1-P5). Inter-group comparisons of normalized EMG were performed using Statistical Parametric Mapping (SPM). The root mean square (RMS) amplitude of each muscle per phase was compared between groups using t-tests. Spearman correlation analysis was employed to explore the relationships between muscle RMS and stroking performance.

**Results:**

No significant inter-group differences were found in stroking performance parameters or swing phase durations (p > 0.05). SPM analysis revealed that Level 1 athletes exhibited lower triceps brachii activation during the 80%–87% swing cycle (p = 0.022), higher pronator teres activation during 70%–72% (p = 0.032), higher flexor carpi ulnaris activation during 78%–86% (p = 0.017), and lower extensor carpi radialis brevis activation during 56%–64% (p = 0.019) compared to Level 2 athletes. The RMS comparisons at the uncorrected α = 0.05 level showed that Level 1 athletes had lower biceps brachii activation in P2 (p = 0.028), higher flexor carpi ulnaris activation in P4 (p = 0.011), and lower triceps brachii activation in P4 (p = 0.044) than Level 2 athletes. Exploratory correlation analyses revealed nominal associations (uncorrected p < 0.05) between flexor carpi ulnaris activation during P2 and carry distance (ρ = 0.528), biceps brachii activation during P2, P3 and backspin (ρ = 0.574, 0.575), and pronator teres activation during P3 and angle of attack (ρ = 0.592).

**Conclusion:**

The activation of the flexor carpi ulnaris, pronator teres, and other follower arm muscles exhibited temporal specificity and showed nominal associations with stroking outcomes. Skill-level differences were observed in both the SPM and RMS of muscle activation, with the most pronounced distinctions occurring in phase specific patterns during the downswing and follow through. The mechanical mechanisms underlying these activation patterns and their functional relevance to the stroking performance remain uncertain, warranting further investigation with synchronized kinematic and kinetic measurements.

## 1 Introduction

As the longest and lightest club in a golfer’s bag, the driver’s performance directly determines the strategic starting point for each hole (Parker et al., 2017). PGA Tour reveals that scoring differences on par-4 holes primarily stem from the distance and accuracy achieved with the driver. A powerful tee shot creates an advantage for the subsequent approach shot to the green, thereby creating scoring opportunities (James, 2017). Optimal driver performance results from the dynamic coupling of multiple parameters, including clubhead speed, angle of attack, launch angle, spin rate, and ball speed. Clubhead speed directly determines ball velocity and flight distance, while the launch angle and attack angle jointly influence the trajectory pattern. The amount of backspin, in turn, affects flight stability and roll distance upon landing (Dong and Ikuno, 2023; Shaw et al., 2023). At present, analyses of high-level golf swing mechanics primarily focus on the rotational sequencing of the trunk and core, the efficiency of energy transfer along the kinetic chain, and the delayed release and whipping effects of the upper limbs (Mahadas et al., 2019; Zhou et al., 2022; Liu et al., 2025). Upper limb functional roles during driver swings vary noticeably by swing technique and golfer skill level, though consistent biomechanical observations have documented typical contributions of the lead and follower arm segments. The lead arm, also termed the guiding or stabilizing arm, is widely considered to support swing plane maintenance, regulate swing arc radius, and moderate swing path trajectory across most swing styles (Liu et al., 2025). Previous studies have defined the sequential motion of the pelvis, thorax, lead forearm, and club as the kinetic chain (Parker et al., 2017; Liu et al., 2025). The follower arm (non-lead arm; the right arm for right-handed golfers) typically contributes to clubface orientation modulation and timing of distal release acceleration (Bochnia et al., 2024). During the backswing, follower arm elbow adduction and flexion often assists lead arm club initiation and facilitates potential energy storage within the kinetic chain (Fedorcik et al., 2012). During the downswing, coordinated torso rotation paired with follower arm elbow extension can improve energy transmission toward the clubhead (Marta et al., 2016). At impact, the rotational orientation of the trail forearm and the wrist joint angle determine the clubface angle (Lee and Park, 2018). However, the traditional coaching perspective, which simplistically defines the role of the follower arm as passive guidance or stabilization, is increasingly challenged by growing biomechanical and neuromuscular evidence. Farber et al. found the follower arm pronator teres is significantly more active in amateurs than professionals during the forward swing (Farber et al., 2009), while Robinson et al. reported greater extensor carpi ulnaris recruitment in the trail arm during downswing (p < 0.001) (Robinson et al., 2023). These findings suggest the follower arm plays an active role. Nevertheless, gaps remain regarding systematic multi-muscle analysis, fine temporal resolution, performance correlations, and skill-level comparisons. The present study addresses these by analyzing five follower arm muscles across five phases, correlating RMS with performance outcomes, and comparing two skill levels. The golf swing is an explosive, multi-segmental motion requiring full-body coordination, yet whether the follower arm serves a predominantly passive or actively coordinated role remains insufficiently investigated.

Surface electromyography (sEMG) analyses of golf swings have historically prioritized trunk core and lower-limb muscle groups (Sorbie et al., 2018; Li et al., 2023). Existing literature has documented lumbar injury risk during the downswing and follow through, skill dependent pelvic rotation driven by hamstring contraction, and distinct lower limb activation timing between professionals and amateurs (Marta et al., 2016; Lee and Park, 2018; Lim et al., 2012). Upper extremity injuries can also trigger full body compensatory muscle activation, disrupting swing efficiency (Ozaki et al., 2016). Despite abundant data on the trunk and lower body, few golf EMG studies systematically quantify phase resolved activation across multiple follower arm muscles or link trail limb neuromuscular activity to key driving performance indicators. Broader cross-sport sEMG research on unilateral striking, throwing, and racquet movements collectively demonstrates forearm flexors are not purely passive stabilizers; precise modulation of activation magnitude, timing, and muscle synergy mediates distal power transfer and control (Hazari and Warsi, 2015; Matsunaga and Kaneoka, 2018; van Trigt et al., 2021; Loushin et al., 2022). Supplementary evidence further confirms upper extremity neuromuscular profiles shift markedly with grip orientation, contraction mode, and interlimb motor asymmetry, illustrating universal task-specificity in limb muscle recruitment patterns (Çuvalcioğlu et al., 2020; Devrilmez et al., 2026; Uysal et al., 2026). Critical biomechanical distinctions limit direct translation of these unilateral sport findings to golf, while the driver swing demands tight bilateral coordination of lead and follower-arm segments. A small body of golf-specific upper limb sEMG work has confirmed skill level differences in trail forearm pronator and wrist flexor activation, yet these prior studies lack multi-muscle profiling across standardized swing phases and fail to quantify statistical associations between follower arm activity and hitting outcomes. This unresolved gap motivates the present multi-muscle, phase resolved sEMG investigation of follower arm function across elite and sub-elite golfers.

Existing investigations of the upper limbs during the golf swing has primarily focused on kinematics, utilizing high-speed video and three-dimensional motion capture technologies to precisely quantify the angles, angular velocities, and movement trajectories of the shoulder, elbow, and wrist joints. This includes key kinematic features such as shoulder external rotation angle at the top of the backswing, the phenomenon of “delayed release” of the elbow during the downswing, and the whip-like speed of the wrist at impact (Williams et al., 2022). Existing EMG studies indicate that the wrist flexor muscles (flexor carpi ulnaris and flexor carpi radialis) exhibit activation levels exceeding 100% MVC during the downswing and impact phases. Values exceeding 100% MVC should be interpreted with caution, as they may arise from methodological factors (e.g., dynamic vs. static testing differences, electrode movement, or MVC familiarization) rather than indicating genuine supra-maximal activation. This finding suggests that these muscles play a significant role in the swing, yet the underlying mechanisms remain unexplored (Fedorcik et al., 2012). Furthermore, research on the muscle activity of the follower arm in golf remains notably insufficient, particularly studies that systematically quantify the relationship between the activation characteristics of its specific muscle groups and key physical parameters of driver performance. Research Hypothesis: Contrary to the prevailing notion in the field that the follower arm “should not actively exert force,” we hypothesize that the neuromuscular activity of specific muscle groups, especially those controlling forearm pronation and wrist flexion, exhibits significant and specific correlations with precision-related parameters such as the angle of attack and backspin. Accordingly, we propose the following three hypotheses: (1) normalized EMG time series during the driver swing differ between Level 1 and Level 2 athletes, as assessed by Statistical Parametric Mapping; (2) phase-averaged RMS amplitudes of the five follower-arm muscles differ between the two skill groups; and (3) phase-specific RMS amplitudes are significantly associated with key stroking performance parameters, including clubhead speed, carry distance, launch angle, angle of attack, and backspin. To test these hypotheses, the present study employed sEMG to record muscle activity from the follower-side triceps brachii, biceps brachii, pronator teres, flexor carpi ulnaris, and extensor carpi radialis brevis during driver swings performed by male golfers of two skill levels. The analysis will focus on comparing muscle activation differences between skill levels and conducting correlation analyses with driver stroking performance parameters to explore the associations between follower arm muscle activity and swing outcomes.

## 2 Materials & methods

### 2.1 Participants

Sixteen right-handed golf athletes with the qualifications of National Level 1 or Level 2 athletes (see Table 1) from Tianjin University of Sport were recruited as participants for sEMG signal and shot performance data collection during driver swing. No *a priori* power analysis was performed. A *post hoc* sensitivity analysis using G*Power 3.1 (α = 0.05, power = 0.80, two-tailed) indicated that, with n = 8 per group, the minimum detectable effect size is Cohen’s d = 1.06 for between-group comparisons and ρ = 0.60 for Spearman correlations (N = 16). Thus, the study is powered only to detect large effects. The present findings should therefore be considered exploratory, warranting confirmation in larger cohorts. Inclusion criteria were: (1) No history of severe upper limb, spinal, or lower limb musculoskeletal injuries requiring medical intervention in the preceding 6 months (2) Restricted resistance exercise, or aerobic endurance activity for a full 24 h before laboratory testing. (3) Consistent regular golf training volume maintained for at least three consecutive months before data collection (4–6 sessions a week). (4) On the day of the test, the subject had no acute fatigue, fever, muscle aches, joint pain, or neuromuscular discomfort. This study was approved by the Ethics Committee of Tianjin University of Sport (TJUS2024-062). The recruitment period for this study is from 2024/12/30 to 2025/01/20. All data collection was completed during this period to ensure that all evaluations were conducted within the same testing window. All participants signed informed consent forms.

**TABLE 1:** Basic information of golfers.

Indicators	Level 1 (n = 8)	Level 2 (n = 8)	t	p	Effect size
Height (m)	1.82 ± 0.06	1.79 ± 0.07	0.765	0.457	0.382
Body Mass (kg)	78.13 ± 11.81	80.52 ± 16.63	−0.31	0.761	0.155
Age (year)	18.75 ± 1.09	20.13 ± 1.54	−1.932	0.074	0.966
Golf experience (year)	6.19 ± 2.42	7.13 ± 3.62	−0.569	0.578	0.285
Handicap*	1.44 ± 1.18	5.50 ± 2.01	−4.615	<0.001	2.307

The National Level 1 and Level 2 classifications are part of the Chinese Athletic Level system administered by the General Administration of Sport of China. Level 1 athletes must have placed in the top two in individual or team competitions at the provincial level or higher, while the Level 2 athletes must have placed third through eighth in individual competitions or third through fourth in team competitions at the provincial level or higher. * indicates a statistically significant difference between Level 1 and Level 2 (p < 0.05).

### 2.2 Experimental protocol

Surface Electromyography Measurement: A YW-Wireless wireless sEMG system (Zhiyunwei, Beijing Changfeng Technology Co., Ltd., China) was used, with five sensor units attached to the right upper extremity muscles of participants: triceps brachii (lateral head), biceps brachii (long head), pronator teres, flexor carpi ulnaris, and extensor carpi radialis brevis. Disposable bipolar AgCl surface electrodes were applied following SENIAM recommendations. The inter-electrode centre-to-centre distance was set to 20 mm, and electrodes were aligned parallel to the predominant muscle fibre direction. Prior to attachment, the target skin region was cleaned with alcohol wipes, lightly abraded, and dried to reduce skin impedance. A shared reference electrode was placed over the bony olecranon process of the elbow. To mitigate crosstalk between adjacent forearm musculature, electrodes were positioned strictly within the mid-muscle belly, avoiding muscle boundaries and tendinous regions. Care was taken to maintain consistent placement across all participants. The sampling frequency was set to 1,000 Hz. Motion Capture Measurement: A Qualisys motion capture system (Qualisys Track Manager, Qualisys AB, Sweden) was employed based on the Qualisys Sports Marker Set-golf model. Thirty-five 16-mm infrared reflective markers were attached to anatomical landmarks on the human body (see Figure 1A), and six markers were affixed to the golf club. The system recorded the full swing motion, divided key swing events, and calculated the swing cycle, with a sampling frequency of 250 Hz. Synchronization: An external repeater was used to achieve external synchronization between the motion capture system and the wireless sEMG system. A shared digital pulse simultaneously triggered data acquisition on both recording devices. The manufacturer specifies temporal synchronization precision <1 m. Pre-experiment calibration tests revealed no systematic temporal offset between signals; therefore, *post hoc* time shift correction was not applied. Stroking Performance Measurement: A 3.15 m × 1.6 m × 2.8 m hitting cage was constructed in the laboratory. A golf hitting mat was placed at a distance of ≥4.5 m from the cage, and participants placed the golf ball on a rubber tee with a height of 0.055 m for hitting. A Flight Scope Xi launch monitor (FlightScope Inc., Orlando, FL, United States of America) was positioned directly behind the ball (≥3 m from the ball position) to collect shot performance data. The launch monitor employed high-speed Doppler radar to record instantaneous ball velocity, spin rate, launch angle and ball flight vectors; carry distance is calculated using built-in aerodynamic models instead of direct measurement of full outdoor ball flight. Although the enclosed hitting cage prevents unobstructed full ball flight, all impact phase kinematic variables were acquired independently of subsequent flight trajectory. Published laboratory validations support reliable measurement of impact related radar outputs within indoor constrained environments. To balance experimental control with ecological validity, all participants used the same model of golf ball (Titleist Pro V1, United States of America) and the same tee height (0.055 m) to minimize ball-related and setup variability. However, participants used their own drivers and golf shoes, as this maximizes their competitive performance by allowing them to execute swings in their most comfortable, well-adapted state. Prior to swing data collection, all participants performed standardized maximal voluntary contraction (MVC) trials for each of the five target muscles following the 10 min warm up and 10 practice swings. For each muscle, participants performed three 5-s maximal isometric contractions against manual resistance provided by the investigator, with 60 s of rest between contractions. The highest RMS value among the three trials was used for subsequent EMG normalization. MVC testing was conducted before swing data collection to obtain true maximal EMG reference values in a non-fatigued state, thereby avoiding the underestimation of MVC that would occur if testing were performed after multiple maximal-effort swings. A 5-min rest period was provided after MVC testing before the familiarization swings began. Participants were then allowed up to five familiarization swings with full effort to acclimate to the laboratory setting and equipment. Following familiarization, 2 min rest period was provided. Each participant then performed at least three full swing strokes, resting for at least 30 s between each swing to minimize the effects of fatigue. All swings were recorded simultaneously by the motion capture system, sEMG system, and launch monitor. A swing was accepted for analysis if it met the following criteria: (1) the ball made solid contact with the center of the clubface (confirmed by launch monitor data), (2) launch direction deviation ≤ ±5° from the target line, (3) no visible swing artifacts, and (4) all motion capture markers remained visible throughout the swing.

**FIGURE 1:**
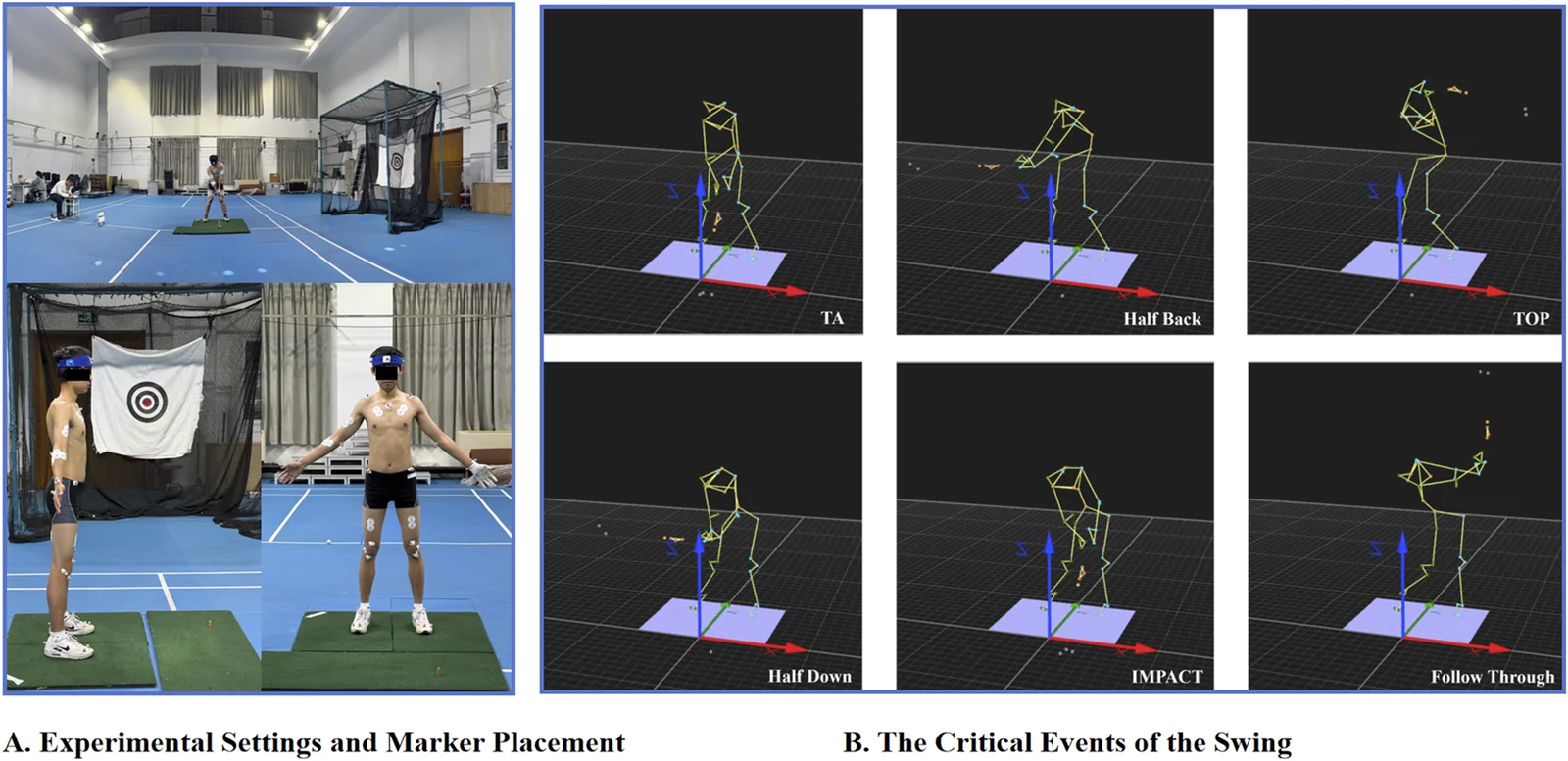
Experimental settings and swing events. **(A)** Experimental setting and marker placement. **(B)** The critical events of the swing.

### 2.3 Variable definitions

Swing Events: All kinematic calculations were conducted in the Qualisys global laboratory coordinate system. The club shaft vector was reconstructed using four markers mounted along the shaft and two markers attached to the clubhead. Take Away (TA) denotes the instant when clubhead linear velocity magnitude first exceeds 0.1 mph; this threshold eliminates trivial pre-swing oscillations to identify the onset of intentional motion. Half Back corresponds to the backswing frame where the shaft vector lies horizontal within ±1.5° relative to the global horizontal plane during clockwise motion away from the ball. Top of Backswing (TOP) is the moment when shaft angular velocity about the vertical axis equals zero. Half Down represents the downswing frame satisfying the same ±1.5° horizontal shaft criterion during counterclockwise motion toward the ball. Impact (IMPACT) is defined as the frame immediately before ball contact, detected by an abrupt shift in clubhead marker velocity. Follow Through maximum height refers to the time of the maximum vertical coordinate of the clubhead markers post-impact (see Figure 1B). Swing Phases: Phase 1 (P1, Take Away Phase): Encompasses the period from TA to Half Back; Phase 2 (P2, Backswing Phase): Spans from Half Back to TOP; Phase 3 (P3, Downswing Release Phase): Covers the interval from TOP to Half Down; Phase 4 (P4, Acceleration-Impact Phase): Extends from Half Down to IMPACT; Phase 5 (P5, Follow-Through Phase): Includes the period from IMPACT to Follow Through. Stroking Performance: All parameters were derived from the FlightScope Xi launch monitor. Clubhead Speed: The maximum linear velocity of the clubhead immediately prior to impact; Carry Distance: The straight-line distance between the original ball position and the first landing point of the ball; Launch Angle: The angle between the ball’s flight direction and the horizontal plane of the launch monitor; Attack Angle: The angle formed by the clubhead’s movement direction and the horizontal plane; Backspin Rate: The rotational speed of the ball around its horizontal axis. sEMG Variables: Normalized EMG (%MVC): Prior to swing data collection, participants performed MVC trials for the target muscles. For each muscle, three 5-s maximal isometric contractions were performed against manual resistance with 60 s rest between trials, accompanied by strong verbal encouragement, and the highest RMS value was used for normalization. Specific testing positions: triceps brachii (lateral head) with shoulder and elbow flexed at 90° resisting forearm extension; biceps brachii (long head) with shoulder flexed 45° and elbow flexed 90° in supination resisting elbow flexion; pronator teres with elbow flexed 90° and forearm neutral resisting pronation; flexor carpi ulnaris with forearm supinated and wrist neutral resisting wrist flexion with ulnar deviation; and extensor carpi radialis brevis with forearm pronated and wrist neutral resisting wrist extension with radial deviation. For each participant, Root Mean Square (RMS) values were averaged across all valid swings to obtain a single participant-level value, which served as the unit of analysis for between-group comparisons and correlations (Table 2).

**TABLE 2:** Muscle activity RMS (%MVC) of the follower arm.

Indicators	Level 1	Level 2	t/z	p	95% CI	Effect size
P1-triceps brachii	3.72 ± 1.96	3.93 ± 1.49	−0.239	0.815	[-2.08,1.66]	0.120
P1-biceps brachii	7.44 ± 3.13	15.87 ± 10.93	−2.097	0.055	[-17.05,0.19]	1.048
P1-pronator teres	27.97 ± 15.08	24.31 ± 10.33	0.566	0.580	[-10.20,17.52]	0.283
P1-flexor carpi ulnaris	27.24 ± 13.46	24.03 ± 9.09	0.559	0.585	[-9.10,15.52]	0.280
P1-extensor carpi radialis brevis	27.43 ± 15.78	31.13 ± 13.62	−0.503	0.623	[-19.51,12.10]	0.251
P2-triceps brachii**†**	13.08 (5.91, 23.09)	8.32 (5.95, 13.41)	−0.420	0.721	[-9.83,11.12]	0.105
P2-biceps brachii*	5.64 ± 2.37	11.45 ± 6.27	−2.453	0.028	[-10.90,-0.73]	1.226
P2-pronator teres	19.32 ± 11.29	21.61 ± 6.70	−0.493	0.629	[-12.25,7.67]	0.247
P2- flexor carpi ulnaris**†**	16.41 (14.34, 24.86)	19.65 (15.97, 30.68)	0.735	0.505	[-10.50,7.14]	0.184
P2-extensor carpi radialis brevis	29.78 ± 14.50	38.08 ± 9.44	−1.357	0.196	[-21.42,4.82]	0.679
P3-triceps brachii	42.80 ± 19.69	45.36 ± 16.86	−0.279	0.784	[-22.22,17.10]	0.140
P3-biceps brachii	7.22 ± 2.13	8.80 ± 4.78	−0.852	0.415	[-5.55,2.39]	0.426
P3-pronator teres**†**	19.29 (14.20, 26.63)	14.69 (12.34, 23.52)	−0.735	0.505	[-6.10,9.05]	0.184
P3-flexor carpi ulnaris**†**	12.42 (10.13, 20.91)	9.51 (6.31, 12.33)	−1.785	0.083	[-0.90,7.01]	0.446
P3-extensor carpi radialis brevis	27.90 ± 16.37	34.47 ± 11.12	−0.939	0.364	[-21.57,8.44]	0.469
P4-triceps brachii*	42.25 ± 11.99	57.21 ± 14.94	−2.209	0.044	[-29.49,-0.43]	1.104
P4-biceps brachii	15.03 ± 6.49	18.79 ± 10.99	−0.832	0.419	[-13.44,5.92]	0.416
P4-pronator teres	38.44 ± 24.93	48.47 ± 19.19	−0.901	0.383	[-33.87,13.83]	0.451
P4-flexor carpi ulnaris*	26.51 ± 10.66	13.55 ± 6.41	2.944	0.011	[3.52,22.39]	1.472
P4-extensor carpi radialis brevis	34.17 ± 15.52	35.07 ± 9.95	−0.138	0.892	[-14.88,13.08]	0.069
P5-triceps brachii**†**	17.26 (12.62, 46.11)	30.38 (18.08, 44.02)	0.840	0.442	[-20.30,9.50]	0.210
P5-biceps brachii	26.08 ± 18.34	39.81 ± 25.52	−1.236	0.237	[-37.56,10.10]	0.618
P5-pronator teres	32.95 ± 18.43	48.64 ± 14.35	−1.900	0.078	[-33.41,2.03]	0.950
P5-flexor carpi ulnaris**†**	38.42 (10.37, 50.40)	27.05 (20.72, 30.89)	−0.420	0.721	[-13.99,10.85]	0.105
P5-extensor carpi radialis brevis	34.23 ± 13.41	46.93 ± 11.64	−2.022	0.063	[-26.16,0.77]	1.011

### 2.4 Data processing

Raw sEMG signals were processed using custom-written algorithms in MATLAB R2023a (MathWorks, Natick, MA, United States of America). A fourth-order Butterworth band-pass filter with cut-off frequencies of 10–450 Hz was applied to retain the effective frequency band of muscle activity and eliminate noise. All filtering was performed using zero-phase forward-reverse filtering (MATLAB filtfilt function) to preserve temporal alignment relative to swing events. For envelope extraction, the filtered signal was full-wave rectified and then low-pass filtered at 10 Hz (fourth-order Butterworth, zero-phase) to obtain the linear envelope, which reflects the temporal changes in muscle activation intensity and was used for SPM analysis. For RMS analysis, the same band-pass filtered and rectified signals were used, with RMS amplitude calculated using a 200 m moving window with 50% overlap, then averaged within each of the five predefined phases. The envelope signal and RMS values represent complementary analyses: the former was used for continuous time-series waveform comparison between groups (SPM), while the latter was used for phase-specific statistical comparisons of activation intensity. To eliminate inter-individual differences, the sEMG signals recorded during MVC trials were processed using the same envelope extraction and RMS procedures described above. Abnormal Signal Exclusion: sEMG signals were excluded based on objective criteria: saturation in any channel, baseline noise >15 μV RMS, movement artifacts (>5 × mean amplitude in a 100 m window) in ≥2 channels, or signal flatline >100 m. Exclusion was performed blind to group assignment. Of 80 recorded swings, 33 were excluded (10 due to off-center contact, 12 for launch direction >±5°, six for artifacts, and five for marker occlusion), with exclusions balanced between groups (Level 1: 8; Level 2: 9). Final valid samples were 24 swings for Level 1 and 23 for Level 2.

### 2.5 Statistical analysis

All statistical analyses were performed using SPSS 26.0 (IBM Corp., Armonk, NY, United States of America) and SPM1D v0.4.8 (https://spm1d.org/) with a baseline significance level set at α = 0.05. All analyses adopted the participant as the unit of analysis (n = 16, eight participants per group), whereby RMS amplitudes and continuous EMG time-series data from valid repeated swings were averaged for each individual prior to statistical testing to eliminate pseudoreplication and satisfy independence assumptions. For discrete stroking performance, phase duration variables and phase-specific RMS, Shapiro-Wilk normality tests were conducted prior to group comparison; independent samples t-tests were applied for normally distributed data, and Mann-Whitney U tests were used for non-normal data, with uncorrected α = 0.05 adopted for these exploratory multiple comparisons. For continuous muscle activation waveforms, participant-averaged normalized EMG (%MVC) curves (101 points, 0%–100% of the full swing cycle) were compared between skill groups via two-sample SPM{t} tests. Signal smoothness was automatically estimated from model residuals using the full-width at half-maximum Gaussian kernel, and random field theory was applied to control familywise error rate at α = 0.05, with degrees of freedom fixed at df = 14 for all SPM{t} comparisons. Spearman’s rank correlation analysis was used to examine associations between participant-averaged phase-specific RMS values and swing performance parameters (125 correlations), selected for its suitability for the small exploratory sample. All correlation analyses were exploratory; no specific muscle-phase-performance combinations were prespecified as primary hypotheses prior to data collection.

## 3 Results

### 3.1 Stroking performance and swing timing

No significant differences were observed in the five selected stroking performance variables between Level 1 and Level 2 athletes (all p > 0.05). For swing timing, no significant differences were found between groups for any phase duration (P1-P5, all p > 0.05; Table 3).

**TABLE 3:** Stroking performance parameters and swing phase durations.

Indicators	Level 1	Level 2	t/z	p	95% CI	Effect size
Clubhead speed (mph)	108.80 ± 5.77	106.32 ± 6.83	0.785	0.445	[-4.30, 9.26]	0.393
Carry distance (yards)	252.12 ± 17.76	244.56 ± 19.35	0.814	0.429	[-12.35, 27.48]	0.407
Launch angle (°)	12.88 ± 3.08	11.96 ± 2.78	0.630	0.539	[-2.22, 4.06]	0.315
Angle of attack (°)	5.14 ± 3.21	4.78 ± 3.90	0.201	0.844	[-3.47, 4.20]	0.100
Backspin (rpm)	2671.83 ± 230.06	2934.81 ± 410.37	−1.581	0.136	[-619.73, 93.77]	0.791
P1 duration (s) **†**	0.60 (0.52, 0.72)	0.53 (0.49, 0.62)	−1.261	0.234	[-0.04,0.21]	0.315
P2 duration (s)	0.304 ± 0.067	0.317 ± 0.035	−0.463	0.650	[-0.07,0.05]	0.232
P3 duration (s)	0.156 ± 0.018	0.180 ± 0.037	−1.626	0.126	[-0.06,0.01]	0.813
P4 duration (s)	0.097 ± 0.008	0.093 ± 0.009	0.831	0.420	[-0.01,0.01]	0.416
P5 duration (s) **†**	0.19 (0.16, 0.19)	0.17 (0.15, 0.19)	−0.739	0.505	[-0.07,-0.04]	0.185

Data are presented as mean ± SD, for normally distributed variables and as median (Q1, Q3) for non-normally distributed variables (†). For t-tests, effect size is Cohen’s d and 95% CI, is for the mean difference. For Mann-Whitney U tests, effect size is the Hodges-Lehmann median difference (HL) and 95% CI, is for the median difference.

### 3.2 Normalized EMG

SPM {t} tests identified continuous time intervals exhibiting intergroup differences across the full swing waveform. The swing cycle divisions for each phase were as follows (Figure 2), P1: 0%–48% of the swing cycle for Level 1 athletes, and 0%–43% for Level 2 athletes; P2: 49%–69% of the swing cycle for Level 1 athletes, and 44%–67% for Level 2 athletes; P3: 70%–80% of the swing cycle for Level 1 athletes, and 68%–80% for Level 2 athletes; P4: 81%–87% of the swing cycle for both Level 1 and Level 2; P5: 88%–100% of the swing cycle for both Level 1 and Level 2. As shown in Figure 3, the normalized EMG of the triceps brachii in Level 1 athletes was significantly lower than that in Level 2 athletes during the 80%–87% swing cycle (t = −3.324, p = 0.022). The normalized EMG of the pronator teres was significantly higher than that of Level 2 athletes during the 70%–72% swing cycle (t = 2.170, p = 0.032). The normalized EMG of the flexor carpi ulnaris was significantly higher than that of Level 2 athletes during the 78%–86% swing cycle (t = 2.485, p = 0.017). The normalized EMG of the extensor carpi radialis brevis in Level 1 athletes was significantly lower than that in Level 2 athletes during the 80%–87% swing cycle (t = −2.415, p = 0.019).

**FIGURE 2:**
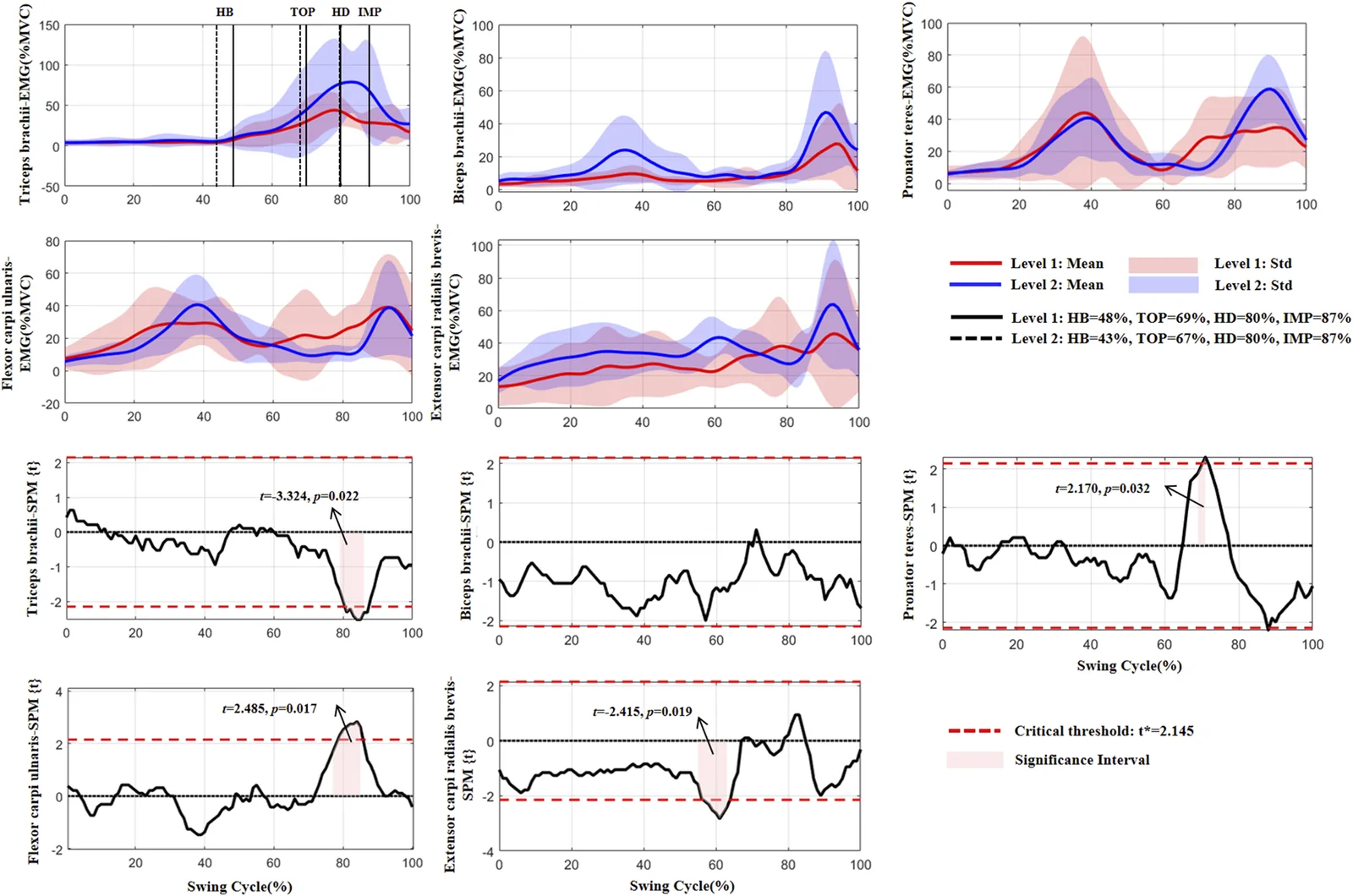
Normalized EMG during the full swing cycle (spm).

**FIGURE 3:**
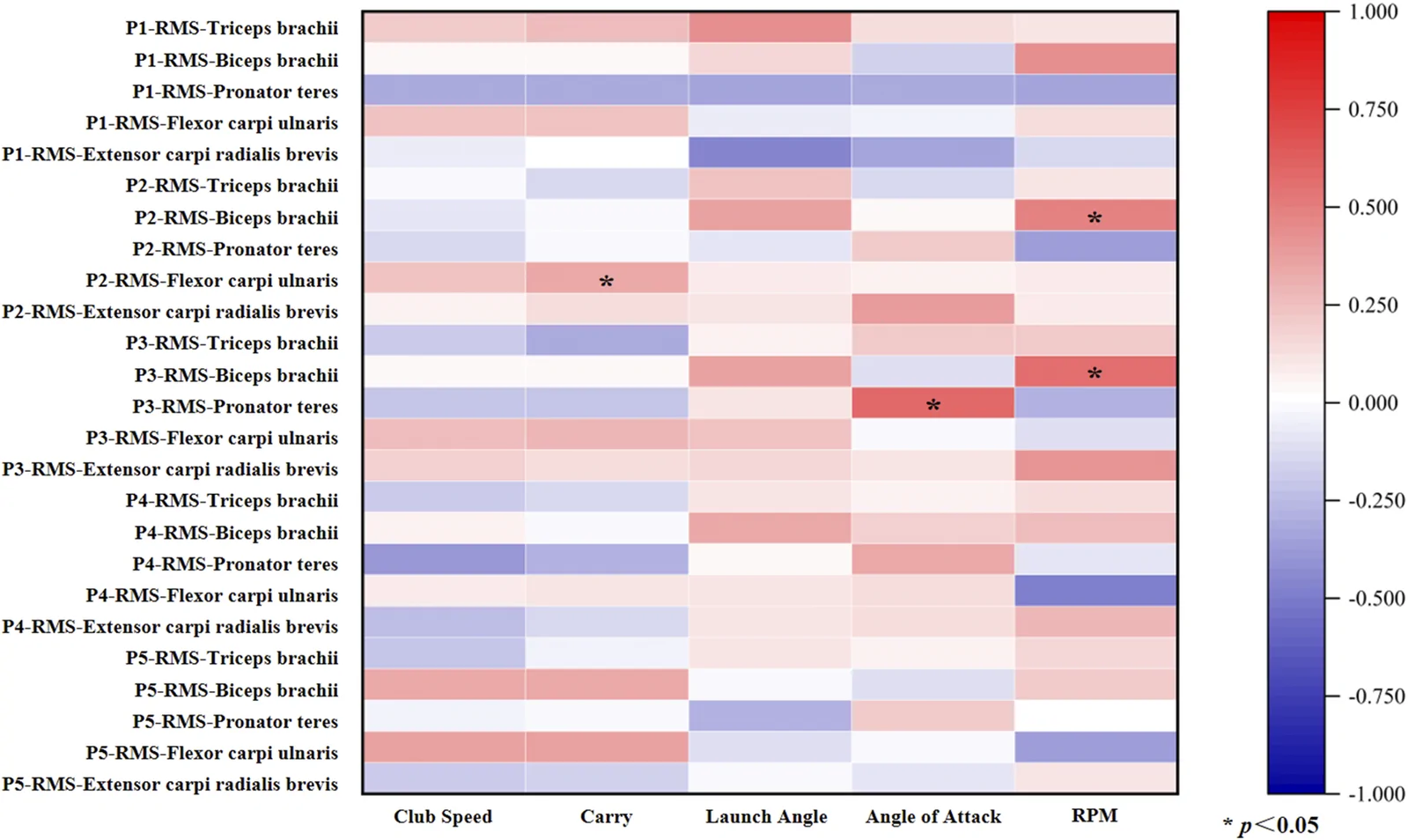
Correlation between Muscle Activity RMS and Stroking Performance Parameters. FDR correction (Benjamini–Hochberg, q = 0.05) was applied across all 125 correlations; all adjusted q-values were >0.05, indicating that no correlation survived correction for multiple testing. Uncorrected p-values are shown for transparency (*p < 0.05) and should be interpreted strictly as nominal associations, not as confirmatory findings.

### 3.3 RMS

The phase-specific RMS comparisons quantified average activation magnitude within discrete, functionally relevant swing phases. This metric supports assessment of aggregate muscle activation during key movement stages and enables direct comparisons with prior golf biomechanics studies reporting phase-averaged EMG values. During the P1 phase, the biceps brachii RMS of Level 1 athletes was lower than that of Level 2 athletes (t = −2.097, p = 0.055, d = 1.048, 95% CI [-17.05, 0.19]). In P2, the biceps brachii RMS of Level 1 athletes was lower than that of Level 2 athletes (t = −2.453, p = 0.028, d = 1.226, 95% CI [-10.90, −0.73]). In P3, the flexor carpi ulnaris RMS of Level 1 athletes was higher than that of Level 2 athletes (U = 12.0, p = 0.083, HL = 2.41%, 95% CI [-0.90, 7.01]). In P4, the flexor carpi ulnaris RMS of Level 1 athletes was higher than that of Level 2 athletes (t = 2.944, p = 0.011, d = 1.472, 95% CI [3.52, 22.39]). The triceps brachii RMS of Level 1 athletes was lower than that of Level 2 athletes (t = −2.209, p = 0.044, d = 1.104, 95% CI [-29.49, −0.43]). No other notable differences were observed in P4. In P5, the pronator teres RMS of Level 1 athletes showed a trend toward lower values than Level 2 athletes (t = −1.900, p = 0.078, d = 0.950, 95% CI [-33.41, 2.03]), and the extensor carpi radialis brevis RMS showed a similar trend (t = −2.022, p = 0.063, d = 1.011, 95% CI [-26.16, 0.77]).

### 3.4 Correlation analysis

Correlation analysis was implemented on participant-averaged data pooled across both athlete groups. Biomechanical interpretation prioritises relationships between muscle activation and impact delivery parameters (angle of attack, launch angle, backspin), given follower-arm muscle function directly modulates clubhead orientation at impact. Due to sample size limitations, all analyses in this study were exploratory in nature. As shown in Figure 3, four correlations reached nominal significance (uncorrected p < 0.05), though none survived FDR correction. Carry distance showed a nominal positive association with flexor carpi ulnaris RMS during P2 (ρ = 0.528, p = 0.036, q = 0.375, 95% CI [0.044, 0.811]). Backspin rate showed nominal positive associations with biceps brachii RMS during P2 (ρ = 0.575, p = 0.020, q = 0.278, 95% CI [0.110, 0.833]) and P3 (ρ = 0.574, p = 0.020, q = 0.278, 95% CI [0.093, 0.837]). Angle of attack showed a nominal positive association with pronator teres RMS during P3 (ρ = 0.592, p = 0.016, q = 0.250, 95% CI [0.120, 0.845]).

## 4 Discussion

The golf swing is a highly coordinated neuromuscular explosive movement, manifesting as the result of complex coupling within the full-body kinetic chain. Its biomechanical outcome is collectively determined by the sequential activation and force transmission across multiple segments, including the trunk, lower limbs, and upper limbs (Liu et al., 2025; Crotty et al., 2024). As an important segment within the upper-limb kinetic chain, follower-arm muscle activation patterns may modulate club delivery characteristics, including clubface orientation, angle of attack and ball speed. In the present study, no statistically significant intergroup differences were identified between Level 1 and Level 2 athletes for any measured stroking performance variable (clubhead speed, carry distance, launch angle, angle of attack, backspin rate). Similarly, comparisons of discrete swing phase durations revealed no significant group disparities across P1-P5. Although descriptive mean differences existed for several metrics, these observational trends did not reach the threshold for statistical significance and cannot be interpreted as evidence of systematic performance or temporal strategy divergence between skill cohorts. Tee-shot strategy in competition varies between maximising distance and prioritising positional accuracy; such tactical variation relies on refined neuromuscular control during swing execution (Israely et al., 2018; Jarque-Bou et al., 2024). However, the present dataset did not detect consistent intergroup differences in swing timing or aggregate shot outcomes that would distinguish the two skill groups.

In traditional instruction, the follower arm is viewed as primarily assisting the lead arm in club initiation, with its elbow flexion passively following the lead arm’s motion, and coaches often advise minimizing its active exertion (Farber et al., 2009). The coaching term “passive motion” describes a perceived sequencing relationship and should not be interpreted as an absence of muscle activity. Even when joint kinematics appear driven by contralateral limb movement, follower-arm musculature may still generate active tension to stabilize the elbow, regulate timing, and modulate club delivery. Consistent with this, bilateral electromyography studies have documented discrete, phase-specific activation patterns for both arms, confirming meaningful follower-arm engagement during the downswing and near impact (Robinson et al., 2023). Therefore, reduced normalized EMG amplitude in the follower arm should not be automatically equated with the “passive” behavior described in coaching theory without direct within-participant bilateral comparison between lead and follower limbs. Normalized EMG profiles showed that triceps brachii activation remained low until 60% of the swing cycle before sustaining through completion. Level 1 athletes reached peak triceps activation earlier (78% cycle, 44.04 ± 21.90 %MVC) with lower intensity than Level 2 (84% cycle, 78.53 ± 39.26 %MVC). Normalized biceps brachii activation gradually increased in the latter portion of the swing cycle, with sustained activity evident from approximately the 80% swing cycle until movement completion. Level 2 athletes attained peak activation at the 91% swing cycle (46.81 ± 37.66 %MVC), ahead of Level 1 athletes, who peaked at the 94% swing cycle (27.75 ± 23.57 %MVC). Normalized pronator teres activation demonstrated divergent temporal patterns. Level 1 athletes reached an early activation peak at the 38% swing cycle (44.01 ± 47.76 %MVC), after which activity decreased and stabilized. Level 2 athletes presented an initial reduction in activation followed by a secondary increase, with peak activity occurring later at the 90% swing cycle (58.79 ± 21.20 %MVC). Normalized flexor carpi ulnaris showed divergent activation waveforms. Level 1 athletes maintained continuous muscle activity with a single peak at 93% swing cycle (39.09 ± 32.57 %MVC). Level 2 athletes displayed bimodal activation, featuring two activity bursts between 30%–40% and 90%–100% swing cycle, with the maximal activation observed at the 38% swing cycle (40.64 ± 18.56 %MVC). Normalized extensor carpi radialis brevis reached peak activation at the 93% swing cycle in both groups. Level 2 athletes demonstrated a higher peak activation (63.75 ± 39.82 %MVC) relative to Level 1 athletes (45.80 ± 45.53 %MVC). The present study revealed phase-specific differences in follower arm muscle activation between Level 1 and Level 2 golfers. In P1-P2, Level 1 athletes exhibited lower biceps brachii activation than Level 2 (P1: p = 0.055, d = 1.048; P2: p = 0.028, d = 1.226), suggesting potential differences in upper-limb coordination during the takeaway and backswing. In P3-P4, Level 1 athletes showed higher flexor carpi ulnaris activation (P3: p = 0.083; P4: p = 0.011, d = 1.472) and lower triceps brachii activation (P4: p = 0.044, d = 1.104) than Level 2. While the higher flexor carpi ulnaris activation is consistent with its known role as a wrist stabilizer during gripping tasks, wrist kinematics and joint kinetics were not directly measured. In P5, Level one athletes showed lower pronator teres (p = 0.078, d = 0.950) and extensor carpi radialis brevis activation (p = 0.063, d = 1.011), which could reflect faster post-impact muscle reduction, but may also arise from differences in grip relaxation or stabilization demands. Importantly, no significant group differences were found in stroking performance (all p > 0.05), cautioning against interpreting EMG differences as superior or inferior movement quality. Future studies incorporating kinematic, kinetic, and bilateral EMG measurements are needed to determine the functional significance of these activation patterns.

Exploratory correlation analysis was conducted to examine phase-specific associations between follower arm muscle activation and swing performance parameters. Notably, all observed relationships reached only nominal significance (p < 0.05) and failed FDR correction, indicating that these trends should be interpreted with extreme caution and cannot support definitive functional or mechanistic conclusions. Consistent with statistical limitations, all interpretations in the present study are descriptive and non-causal, as correlation analysis cannot verify directional modulation or biomechanical causation. Nominal exploratory trends indicated that P2 flexor carpi ulnaris activation was positively associated with carry distance. Although the flexor carpi ulnaris serves a stabilising role in wrist biomechanics, the present dataset lacks direct measurements of wrist stiffness, joint torque, and intersegment energy transfer (Durand et al., 2019). Accordingly, this association cannot be attributed to enhanced wrist stability or improved kinetic chain transmission and merely represents a tentative statistical covariation observed in the current sample. P2 and P3 biceps brachii activation showed nominal positive correlations with backspin rate, while P3 pronator teres activation was tentatively associated with an increased angle of attack (Lee et al., 2024). These phase-specific trends suggest potential temporal coupling between upper-limb muscle activity and impact delivery variables across swing stages (Koizumi et al., 2023). Since muscle co-contraction indices, club-ball contact mechanics, and impact load characteristics were not quantified, the present EMG data cannot explain the physiological or mechanical basis for observed backspin-related trends. Similarly, tentative associations between late-phase forearm muscle activity and reduced backspin do not warrant inference of improved wrist stability or cleaner impact contact (Cho and Song, 2021). Overall, the current correlational outcomes do not demonstrate robust, statistically adjusted relationships between follower-arm muscle RMS and swing performance. No systematic, functionally deterministic activation patterns were identified. All observed nominal trends are exploratory and sample-dependent. Future work integrating synchronized kinematic, kinetic, and contact mechanical measurements is required to validate whether these tentative sEMG performance covariations reflect meaningful neuromuscular control strategies during golf swing execution (Manocha et al., 2017).

### 4.1 Limitations and prospects

Several methodological limitations should be considered when interpreting the present findings. The sample size was modest (n = 8 per group), with no *a priori* power analysis; *post hoc* sensitivity analysis indicated the study was powered only to detect large effects, and correlations were non-significant after FDR correction, supporting an exploratory interpretation. Surface EMG was recorded from only five follower-arm muscles without lead-arm recordings, precluding assessment of bilateral coordination or complete upper-extremity synergies, and deep forearm muscles were not accessible. Multiple statistical comparisons were performed, and no correlations survived FDR correction, reinforcing the hypothesis-generating nature of the findings. The laboratory hitting cage restricted full ball flight, carry distance was estimated via aerodynamic modelling, and participants used their own drivers and shoes, introducing equipment-related variability. The sample comprised exclusively right-handed male golfers with Chinese national qualifications, limiting generalizability. Importantly, wrist kinematics, joint torques, segmental energy transfer, co-contraction, and club-ball contact mechanics were not measured; therefore, any interpretations involving these constructs are speculative and require confirmation in future studies incorporating synchronized kinematic, kinetic, and bilateral EMG measurements in larger, more diverse samples.

## 5 Conclusion

No statistically significant intergroup differences were detected between Level 1 and Level 2 golfers for measured stroke performance variables and swing phase durations. The phase-specific follower arm muscle activation patterns were identified across the swing cycle using continuous SPM analysis and phase-averaged RMS metrics. During early swing phases (P1-P2), Level 1 athletes exhibited lower biceps brachii activation relative to Level 2 athletes. In the late downswing and impact interval (P3-P4), Level 1 athletes demonstrated elevated flexor carpi ulnaris activation alongside reduced triceps brachii engagement. Following impact (P5), descriptive trends indicated lower pronator teres and extensor carpi radialis brevis activation among Level 1 athletes. These group disparities reflect skill-dependent divergence in the timing and magnitude of follower-arm muscle recruitment during coordinated golf swing motion. Exploratory correlation analysis revealed several nominal associations between follower-arm muscle RMS and impact delivery parameters; however, none remained significant after FDR correction. The present findings confirm that golfers of different skill levels adopt differentiated follower-arm neuromuscular activation profiles, even when swing performance metrics are statistically indistinguishable. The mechanical mechanisms underlying these contrasting activation patterns, as well as their functional relevance to impact geometry and ball flight, remain unclear. Further multi-modal measurements incorporating synchronous kinematic, kinetic, and bilateral EMG recordings are required to clarify how follower-arm muscle behaviour interacts with full upper-limb coordination during driver tee shots.

## Data Availability

The original contributions presented in the study are included in the article/supplementary material, further inquiries can be directed to the corresponding author.
